# Therapeutic effects of striatal dopaminergic modulation on idiopathic dystonia and OCD in humans: insights from the striosome hypothesis

**DOI:** 10.3389/fnhum.2025.1621054

**Published:** 2025-08-20

**Authors:** Shinichi Matsumoto, Hideki Shimazu, Satoshi Goto

**Affiliations:** ^1^Department of Neurology, Osaka Neurological Institute, Osaka, Japan; ^2^Department of Pharmacology, School of Pharmacy and Pharmaceutical Sciences, Mukogawa Women’s University, Nishinomiya, Japan; ^3^Center for Drug Discovery and Development Sciences, Research Organization of Science and Technology, Ritsumeikan University, Kyoto, Japan; ^4^Department of Clinical Neuroscience, Institute of Biomedical Sciences, Tokushima University, Tokushima, Japan

**Keywords:** dystonia, obsessive-compulsive disorder, striosome, dopamine D1 receptors, D2 antagonist, dopaminergic treatment

## Abstract

Emerging evidence suggests that striatal striosomes play a key role in the dopaminergic regulation of motor and mental action selection processes, with impairments leading to repetitive stereotyped movements (dystonias), thoughts (obsessions), and behaviors (compulsions). To explore this hypothesis therapeutically, we investigated how idiopathic dystonia and obsessive-compulsive disorder (OCD) respond to a novel dopaminergic treatment using low-dose L-DOPA combined with chlorpromazine (CPZ), which can primarily enhance striosomal D_1_ dopamine receptor (D_1_R) signaling in humans. The therapeutic effects of L-DOPA/CPZ were assessed over 1 year in 26 idiopathic dystonia patients (mean age, 55.9 years; 23.1% male) with OCD. The daily doses of L-DOPA/carbidopa and CPZ-phenolphthalinate were increased stepwise to 50 mg and 5 mg, respectively, three times daily over an 8-weeks period, and then maintained for a year. The severity of dystonia and OCD was evaluated using the Burke-Fahn-Marsden Dystonia Movement Scale (BFMDMS) and Yale-Brown Obsessive-Compulsive Scale (Y-BOCS). At a 1-year follow-up, the BFMDMS and Y-BOCS scores improved by approximately 80% (mean difference, −13.8; 95% CI, −16.9 to −10.6; *P* < 0.0001) and 75% (mean difference, −16.0; 95% CI, −16.1 to −15.8; *P* < 0.0001), respectively, with no specific adverse effects. Thus, low-dose L-DOPA/CPZ provided striking and lasting benefits to patients with idiopathic dystonia and OCD. Our findings indicate that dystonia and OCD may share a common striatal dysfunction due to altered D_1_R signaling in the striosomes. Pharmacologic interventions aimed at modulating striosomal D_1_R signaling could enhance our understanding of the striatal mechanisms involved in the pathophysiology of both dystonia and OCD.

## 1 Introduction

Dystonia is a clinical syndrome characterized by involuntary, sustained, or repetitive contractions of antagonistic muscles, causing patterned movements and postures (Albanese et al., 2013). This neurological condition is among the most disabling movement disorders, though its pathophysiology is still not fully understood (Sciamanna et al., 2022). Of interest is that dystonia often occurs alongside several neuropsychiatric symptoms (Cavallaro et al., 2002; Voon et al., 2010; Barahona-Corrêa et al., 2011; Timmers et al., 2019). Most notably, idiopathic dystonia (Cavallaro et al., 2002; Voon et al., 2010; Barahona-Corrêa et al., 2011) and SGCE myoclonus-dystonia (Timmers et al., 2019) coexist with obsessive-compulsive disorder (OCD), although no association with DYT1 dystonia has been observed (Heiman et al., 2007). OCD is a highly prevalent neuropsychiatric condition characterized by repetitive and stereotyped thoughts, urges, or images (known as obsessions), along with repetitive, stereotyped behaviors and mental acts (known as compulsions) (American Psychiatric Association [APA], 2013). Prevailing evidence indicates that both dystonia and OCD occur due to disrupted interactions between the frontal cortex and basal ganglia (Hollunder et al., 2024). However, it remains ambiguous as to which anatomical substrates within the cortico-basal ganglia circuits contribute to the overlapping symptomatic characteristics associated with deficits in movement and behavioral inhibition in conditions such as dystonia and OCD. Multimodal therapeutic strategies have been employed in the treatment of these often debilitating conditions, yielding a diverse range of outcomes.

Accruing evidence suggests that imbalances in the activity between D_1_ and D_2_-type dopamine receptors (D_1_Rs and D_2_Rs) within the striatal striosome-matrix system contribute to the development of various motor and cognitive disorders of corticobasal ganglia origin, including dystonia and OCD (Amemori et al., 2011; Crittenden and Graybiel, 2011; Graybiel and Matsushima, 2023; Goto, 2025). A long-standing hypothesis highlights the critical role of striatal striosomes in the dopaminergic regulation of the mechanisms that facilitates the selection of specific motor and mental actions (for a comprehensive review, see Ref. Graybiel and Matsushima, 2023). This implies that altered dopamine processing in the striosomes may lead to repetitive stereotyped movements (dystonias), thoughts (obsessions), and behaviors (compulsions) (Amemori et al., 2011; Graybiel and Matsushima, 2023). To support this hypothesis within a therapeutic framework, we here present evidence of a significant and durable response of idiopathic dystonia and OCD to a novel dopaminergic treatment using L-DOPA (a dopamine precursor) combined with chlorpromazine (CPZ, a D_2_ antagonist), which can enhance D_1_R signaling primarily in the striatal striosomes in humans (Matsumoto et al., 2022, 2024; Matsumoto and Goto, 2024; Goto, 2025).

## 2 Subjects and methods

### 2.1 Ethics statement

This study received approval from the Institutional Ethics Committee at the Osaka Neurological Institute (reference number: OR04-3), and written informed consent was obtained from all participants involved in the research. Furthermore, it is registered with the UMIN Clinical Trials Registry (UMIN000027430), which is recognized by the International Committee of Medical Journal Editors.

### 2.2 Patient sorting, clinical evaluations, and drug administration

**FIGURE 1:**
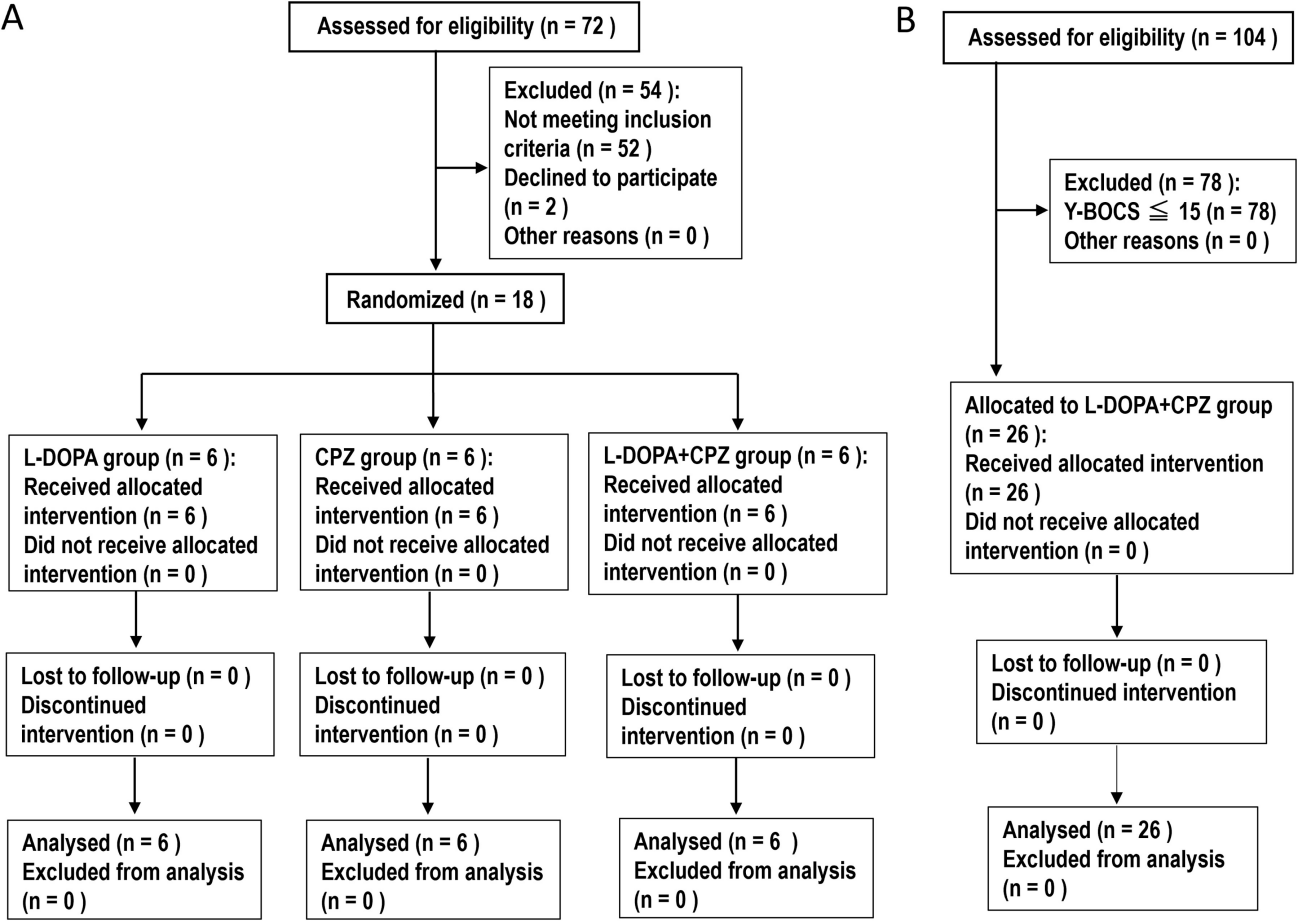
Study Design. **(A)** An 8-weeks follow-up study. This study involved the enrollment of patients with idiopathic dystonia and OCD. A total of 18 participants were randomly assigned by S. M. and categorized into three distinct groups: L-DOPA (*n* = 6), chlorpromazine (CPZ) (*n* = 6), and a combined L-DOPA + CPZ group (*n* = 6). Over an 8-weeks period, the daily doses of L-DOPA/carbidopa and/or CPZ-phenolphthalinate were systematically increased, reaching a maximum of 50 mg administered three times daily and/or 5 mg administered three times daily, respectively. The study groups consisted of participants who received L-DOPA/carbidopa alone, CPZ-phenolphthalinate alone, and a combination treatment of L-DOPA/carbidopa alongside CPZ-phenolphthalinate. Clinical evaluations and video recordings were conducted at baseline (1 day before treatment initiation) and subsequently at 4 weeks and 8 weeks post-treatment commencement. **(B)** A 1-year follow-up study. This study involved a cohort of 26 patients with idiopathic dystonia and obsessive-compulsive disorder (OCD), as indicated by a Yale-Brown Obsessive Compulsive Scale (Y-BOCS) score of 16 or higher. The administration of L-DOPA/carbidopa in conjunction with CPZ-phenolphthalinate was initiated at low dosages and subsequently adjusted in a methodical manner over an 8-weeks period, ultimately achieving doses of 50 mg administered three times daily and 5 mg administered three times daily, respectively. Following this titration phase, the medication dosages were maintained at a constant level until the study’s conclusion, which included a 1-year follow-up period. Clinical assessments were conducted at baseline, as well as at 4 weeks, 8 weeks, and 1 year following the initiation of treatment with L-DOPA/CPZ.

The study design is illustrated in Figure 1. This study enrolled patients diagnosed with both idiopathic dystonia and OCD. The diagnosis of idiopathic dystonia was based on the criteria established by Albanese et al. (2013). The severity of dystonia was evaluated using the Burke-Fahn-Marsden Dystonia Movement Scale (BFMDMS) (Burke et al., 1985). The participants exhibited dystonia symptoms affecting various parts of their bodies (Supplementary Tables 1, 2). Genetic tests and brain MRIs were conducted to exclude hereditary and secondary dystonias. All participants tested negative for disease-causing variations in “currently” known dystonia genes identified by whole-exome sequencing (OMIM Phenotypic Series PS128100).^1^ The patients identified as having idiopathic dystonia were also assessed using the clinician-rated Yale-Brown Obsessive-Compulsive Scale (Y-BOCS), (Goodman et al., 1989) with 10-item scale where each item is rated from 0 to 40. A score of 0–7 is considered subclinical, 8–15 is mild, 16–23 is moderate, 24–31 is severe, and 32–40 is extreme (Goodman et al., 1989). The subtypes of OC symptoms, as categorized by the Y-BOCS Symptom Checklist, are presented in Supplementary Tables 3, 4. Throughout the studies described below, the participants’ concurrent medications, except for L-DOPA and CPZ, remained unchanged (Supplementary Tables 1, 2). Clinical evaluations and video recordings were conducted 1 day before (baseline), at 4 and 8 weeks, and 1 year after initiating the treatment with L-DOPA and/or CPZ.

#### 2.2.1 An 8-weeks follow-up study

In the order of visits, 18 participants were randomly assigned (S. M.) and divided into three distinct groups: L-DOPA, CPZ, and L-DOPA + CPZ groups, as illustrated in Figure 1 and detailed in Supplementary Table 1. In a single-blind study design, participants were not informed of their group assignments. The administration of the drugs was conducted in accordance with the methods that we previously reported (Matsumoto et al., 2022, 2024; Matsumoto and Goto, 2024). Dopacol tablets L50™, containing L-DOPA (50 mg) and carbidopa (5 mg) (Nichi-Iko Pharmaceutical Co., Toyama, Japan), as well as Wintermin™ fine granules (10%; 180 mg of CPZ-phenolphthalinate per gram) (Shionogi Co., Osaka, Japan), were used.

The L-DOPA group comprised 6 participants who received L-DOPA/carbidopa (50 mg/day) for the first 2 weeks, followed by L-DOPA/carbidopa (50 mg twice daily) for the subsequent 2 weeks, and L-DOPA/carbidopa (50 mg three times daily) for the final 4 weeks. The CPZ group consisted of 6 participants, who ingested CPZ-phenolphthalinate (5 mg/day) for the first 2 weeks, followed by CPZ-phenolphthalinate (5 mg twice daily) for the next 2 weeks, and ultimately, CPZ-phenolphthalinate (5 mg three times daily) for the final 4 weeks. The L-DOPA + CPZ group included 6 participants who received L-DOPA/carbidopa (50 mg/day) in conjunction with CPZ-phenolphthalinate (5 mg/day) for the initial 2 weeks, L-DOPA/carbidopa (50 mg twice daily) alongside CPZ-phenolphthalinate (5 mg twice daily) for the following 2 weeks, and L-DOPA/carbidopa (50 mg three times daily) combined with CPZ-phenolphthalinate (5 mg three times daily) over the final 4 weeks.

#### 2.2.2 A one-year follow-up study

This study enrolled a total of 26 patients with idiopathic dystonia and OCD (Y-BOCS ≥ 16) (see Figure 1 and Supplementary Table 2). As outlined in the protocol for the 8-weeks follow-up study, the daily doses of L-DOPA/carbidopa and CPZ-phenolphthalinate were incrementally increased to 150 mg and 15 mg, respectively, over the 8-weeks period. Following this, the medication dosages remained constant throughout the study, which included a 1-year follow-up period.

### 2.3 Statistical analyses

All values are presented as means ± standard deviation (SD). Non-parametric statistical analyses were conducted using the Friedman test, followed by Dunn’s multiple comparison test for further evaluation. A significance level of *P* < 0.05 was set for this study. The analyses were performed using GraphPad Prism version 7.0, developed by GraphPad Software, located in San Diego, California, USA.

## 3 Results

### 3.1 Low-dose L-DOPA and CPZ exert therapeutic effects synergistically

The therapeutic effects of L-DOPA + CPZ, L-DOPA alone, or CPZ alone were assessed over 8 weeks in 18 patients with idiopathic dystonia and OCD, with a mean age of 61.9 years (SD 15.4) and a mean disease duration of 9.2 years (SD 6.0) (see Table 1).

**TABLE 1:** Clinical summary of an 8-weeks follow-up study.

Group	Total numbers (female)	Age (years)	Disease duration (years)	BFMDMS score			Y-BOCS score		
				Baseline	4 weeks	8 weeks	Baseline	4 weeks	8 weeks
L-DOPA	*N* = 6 (4)	67.3 (9.7)	11.5 (7.2)	15.3 (3.0)	15.9 (3.2)	15.9 (3.2)	17.3 (3.2)	19.0 (4.0)	19.5 (8.3)
CPZ	*N* = 6 (5)	50.2 (12.3)	7.0 (5.0)	14.8 (2.8)	15.8 (4.2)	15.8 (3.0)	21.5 (8.4)	19.3 (7.0)	23.6 (8.6)
L-DOPA + CPZ	*N* = 6 (4)	68.3 (17.7)	9.2 (5.6)	**15.3 (4.8)**	**9.6 (2.1)**	**2.8 (2.5)** ***	**19.5 (4.4)**	**10.0 (4.0)**	**5.5 (3.8)** **

In the groups administered L-DOPA (*n* = 6), chlorpromazine (CPZ) (*n* = 6), and L-DOPA combined with CPZ (*n* = 6), the daily doses of L-DOPA/carbidopa and/or CPZ-phenolphthalinate were incrementally increased to a maximum of 50 mg three times daily and/or 5 mg three times daily, respectively, over the course of 8 weeks (see Subjects and methods). Clinical assessments were conducted 1 day prior to the initiation of treatments (baseline), as well as at 4 weeks and 8 weeks post-treatment commencement. The severity of dystonia and OCD was evaluated using the Burke-Fahn-Marsden Dystonia Movement Scale (BFMDMS) and the Yale-Brown Obsessive-Compulsive Scale (Y-BOCS), respectively. All values were reported as means (±SD). Statistical significance was determined at **P < 0.01;

***P < 0.001 (*n* = 6; Friedman test with Dunn’s *post-hoc* test) in comparison to baseline values. L-DOPA+CPZ group showing statistical significance at 8 weeks are indicated in bold.

At an 8-weeks follow-up, the combined administration of L-DOPA/carbidopa (50 mg three times daily) with CPZ-phenolphthalinate (5 mg three times daily) resulted in significant improvements in both the BFMDMS and Y-BOCS scores, with reductions of approximately 80% (*n* = 6; mean difference, −12.5; 95% CI, −15.0 to −10.1; *P* = 0.0016) and 70% (*n* = 6; mean difference, −14.0; 95% CI, −14.5 to −13.4; *P* = 0.0016), respectively, when compared to baseline levels. In contrast, the administration of L-DOPA/carbidopa (50 mg three times daily) alone or CPZ-phenolphthalinate (5 mg three times daily) alone did not yield any significant changes in the BFMDMS or Y-BOCS scores from baseline in either the L-DOPA group (*n* = 6) or the CPZ group (*n* = 6).

Thus, medication with low doses of L-DOPA and CPZ is superior to either one alone, suggesting that low-dose L-DOPA and CPZ synergistically act to produce effective therapeutic results in treating idiopathic dystonia and OCD. This notion corroborates our previous reports on patients with idiopathic blepharospasm (Matsumoto et al., 2022) and cervical dystonia (Matsumoto et al., 2024).

### 3.2 Low-dose L-DOPA/CPZ exerts long-lasting therapeutic effects

The therapeutic effects of L-DOPA/CPZ were assessed over 1 year in 26 patients (23.1% male) with idiopathic dystonia and OCD (Y-BOCS ≥ 16), with a mean age of 55.9 years (SD 16.1) and a mean disease duration of 8.6 years (SD 6.9) (see Table 2).

**TABLE 2:** A 1-year follow-up study on the therapeutic effects of low-dose L-DOPA combined with chlorpromazine (CPZ) in patients with idiopathic dystonia and OCD.

Follow-up period	BFMDMS	Y-BOCS	Y-BOCS	Y-BOCS
	Score	Total score	Obsession score	Compulsion score
Baseline	16.8 (10.6)	21.5 (5.4)	11.7 (2.9)	10.0 (3.4)
4 weeks	7.1 (4.9)**	11.0 (7.0)**	7.1 (4.3)**	4.0 (3.9)****
8 weeks	3.5 (3.4)****	8.3 (7.0)****	5.2 (4.2)****	3.3 (3.8)****
One year	3.0 (2.7)****	5.5 (5.8)****	3.4 (3.7)****	2.2 (2.8)****

This study enrolled 26 patients (23.1% male) with idiopathic dystonia and OCD (Y-BOCS ≥ 16), with a mean age of 55.9 years (SD 16.1) and a mean disease duration of 8.6 years (SD 6.9). In all the participants, the daily doses of L-DOPA/carbidopa and CPZ-phenolphthalinate were increased incrementally, culminating in up to 50 mg administered three times daily and 5 mg administered three times daily, respectively, over a period of 8 weeks. Following this, the drug doses remained unchanged until the end of the study, with a 1-year follow-up. Clinical evaluations were conducted 1 day before (baseline), at 4 weeks, 8 weeks, and 1 year after initiating the L-DOPA/CPZ treatment. The severity of dystonia was assessed using the Burke-Fahn-Marsden Dystonia Movement Scale (BFMDMS) score. The severity of OCD was measured using the Yale-Brown Obsessive-Compulsive Scale (Y-BOCS) Total, Obsession, and Compulsion scores. All values were expressed as means (±SD).

**P < 0.01;

****P < 0.0001 (*n* = 26; Friedman test with Dunn’s *post-hoc* test) compared to the baseline. No significant difference was found in all the scores examined between the 8-weeks and 1-year follow-up periods.

The administration of L-DOPA/CPZ resulted in a significant enhancement of the BFMDMS and Y-BOCS scores among all 26 participants involved in the study. At the 1-year follow-up, utilizing daily doses of L-DOPA/carbidopa (50 mg three times daily) and CPZ-phenolphthalein (5 mg three times daily), the BFMDMS score showed an improvement of approximately 80% (mean difference, −13.8; 95% CI, −16.9 to −10.6; *P* < 0.0001) in comparison to baseline measurements. Concurrently, the Y-BOCS total, obsession, and compulsion scores, improved by approximately 75% (mean difference, −16.0; 95% CI, −15.8 to −16.1; *P* < 0.0001), 70% (mean difference, −8.5; 95% CI, −8.7 to −8.1; *P* < 0.0001), and 80% (mean difference, −7.8; 95% CI, −8.0 to −7.6; *P* < 0.0001), respectively. Importantly, no significant differences were observed in the scores evaluated between the 8-weeks and 1-year follow-up periods. It is plausible that the symptoms of dystonia and OCD were primarily mitigated during the initial 8-weeks treatment phase.

Thus, low-dose L-DOPA/CPZ could exert a highly effective and lasting therapeutic impact on idiopathic dystonia (for example, see Supplementary Videos 1–5) and OCD. During this study, none of the participants reported any specific adverse problems, including gastrointestinal issues.

## 4 Discussion

There is a consensus that alterations in the network activity of the cortico-striato-thalamo-cortical (CSTC) loops play a significant role in the pathogenesis of both dystonia and OCD (Alexander et al., 1986; Hollunder et al., 2024). This study seeks to elucidate the mechanisms underlying the overlapping repetitive and stereotyped symptoms associated with the motor and mental impairments inherent to dystonia and OCD, respectively. We present evidence indicating that low-dose L-DOPA/CPZ can provide significant and enduring therapeutic benefits for individuals with idiopathic dystonia and OCD. Since L-DOPA acts as a D_1_/D_2_ agonist and CPZ functions as a D_2_ antagonist, our findings suggest that targeting D_1_R signaling within the CSTC circuits may offer a promising approach for enhancing the understanding of the pathophysiology of both conditions.

### 4.1 The role of striatal (striosomal) dopamine signaling in the CSTC circuits

Striatal dopamine processing, mediated by D_1_Rs and D_2_Rs, is closely linked to a wide range of motor and mental processes, particularly in the context of reinforcement learning (RL) and decision-making (Graybiel and Matsushima, 2023). D_1_R activation promotes goal-directed behaviors and rewards salience, while D_2_R stimulation inhibits specific responses and regulates stimulus incentive value (Graybiel and Matsushima, 2023). A balanced interaction between D_1_Rs and D_2_Rs is essential for optimizing motor control and cognitive flexibility, facilitating motor and behavioral adaptation informed by prior experiences and anticipated outcomes (Graybiel and Matsushima, 2023). It has significant implications for maladaptive movement and behaviors observed in dystonia and OCD. Interestingly, a recent study in humans has shown that a low dose of L-DOPA or haloperidol (a D_2_ antagonist) can regulate decision thresholds in RL, thereby contributing to the mechanisms underlying the selection of specific mental actions (Chakroun et al., 2023).

**FIGURE 2:**
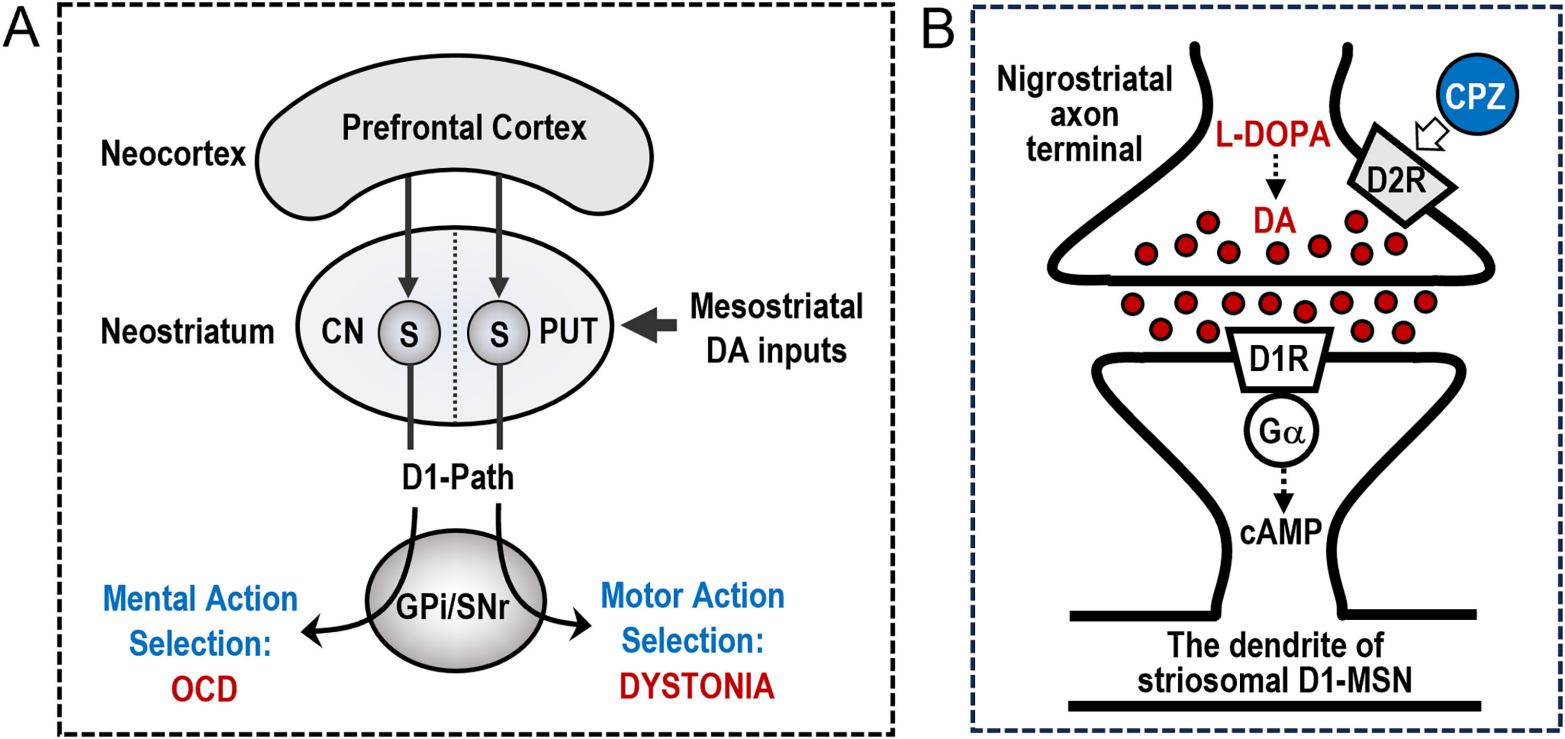
Striosomal D_1_ Dopamine Signaling in Dystonia and Obsessive-Compulsive Disorder (OCD). **(A)** A hypothetical role of the prefronto-striosomal network in dystonia and OCD. The presented diagram provides a simplified representation of the mechanisms by which the cortico-striatal circuit governs the selection of mental and motor actions. Medium spiny neurons that express D_1_-type dopamine receptors (D_1_-MSNs) in the striatal striosomes (S) receive cortico-striatal inputs predominantly from the limbic-associated prefrontal cortex. These D_1_-MSNs constitute the striosomal D_1_ output pathway (D1-Path) to the basal ganglia output nuclei, specifically the globus pallidus internus (GPi) and substantia nigra pars reticulata (SNr). Additionally, striosomal D_1_-MSNs receive dopaminergic signals via the mesostriatal pathway. Within the parallel and interacting cortico-striatal-thalamo-cortical loops, the putamen (PUT) is involved in the sensorimotor circuit, whereas the caudate nucleus (CN) is associated with cognitive functions. Consequently, alterations in the activity of striosomal D_1_-MSNS within the PUT may disrupt specific mechanisms related to “motor” action selection, potentially leading to the development of dystonia. Conversely, changes in the activity of striosomal D_1_-MSNs within the CN may hinder specific mechanisms associated with “mental” action selection, potentially contributing to the manifestation of OCD. **(B)** The therapeutic mechanisms for idiopathic dystonia and OCD. The diagram provided delineates a theoretical model that elucidates the mechanisms through which low doses of L-DOPA and chlorpromazine (CPZ) exert therapeutic effects in treating idiopathic dystonia and OCD. D_1_-type dopamine receptors (D_1_Rs) are heavily enriched in the striosome, but not the matrix, compartment of the human neostriatum. Consequently, the administration of low doses of L-DOPA may selectively affect D_1_R-expressing medium spiny neurons (D_1_-MSNs) situated within the striosomes due to its D_1_ agonistic properties. The activation of D_1_Rs induces an increase in cyclic adenosine monophosphate (cAMP) production via the olfactory-type G-protein α subunit (Gα), thereby enhancing the activity of the striosomal D_1_-MSNs. Moreover, the concurrent use of low doses of CPZ may synergistically enhance the D_1_-agonistic effects of L-DOPA on the activity of striosomal D_1_-MSNs. This phenomenon occurs because low-dose D_2_ antagonists, such as CPZ, primarily target presynaptic D_2_-type dopamine receptors (D_2_Rs), also known as D_2_R autoreceptors, located on the mesostriatal axon terminals. This targeting mechanism facilitates increased dopamine release in the striatum, thereby enhancing overall striatal dopaminergic activity. Therefore, the combined use of low doses of L-DOPA and CPZ represents a novel pharmacological strategy for the treatment of idiopathic dystonia and OCD by augmenting the activity of striosomal D_1_-MSNs.

Mesostriatal dopaminergic inputs are principally integrated by medium spiny neurons (MSNs) expressing D_1_Rs and D_2_Rs (D_1_-MSNs and D_2_-MSNs), which constitute the vast majority of striatal neurons and form the major striatal efferent system integral to the CSTC loops (Crittenden and Graybiel, 2011; Graybiel and Matsushima, 2023). Within the parallel interacting CSTC loops, the putamen participates in the sensorimotor circuit, while the caudate nucleus involves the cognitive circuit (Alexander et al., 1986). Hence, dystonia and OCD may occur due to alterations in dopamine processing in the putamen and caudate nucleus, respectively (see Figure 2A). The striosomes may function as a key regulator of dopamine processing in both the dystonia-associated and OCD-associated CSTC circuits at the striatal level in humans.

### 4.2 The prefronto-striatal (striosomal) network in dystonia and OCD

Repetitive stereotyped motor and cognitive symptoms are key clinical features of both dystonia (Amemori et al., 2011; Crittenden and Graybiel, 2011; Albanese et al., 2013; Matsumoto et al., 2022, 2024; Matsumoto and Goto, 2024; Goto, 2025) and OCD (Amemori et al., 2011; Crittenden and Graybiel, 2011; American Psychiatric Association [APA], 2013; Graybiel and Matsushima, 2023). It has long been suggested that these symptoms may occur due to altered function of a neural circuit that connects the prefrontal cortex to the striosomes, which regulate the dopaminergic control of limbic cortical-striatal circuit processing (Graybiel and Matsushima, 2023). This hypothesis highlights the crucial roles of striosomal D_1_-MSNs in the dopaminergic regulation of RL and decision-making (Graybiel and Matsushima, 2023). Consequently, they contribute to specific motor and mental action selection mechanisms, of which impairments can lead to repetitive stereotyped symptoms in both dystonia and OCD (Figure 2A; Amemori et al., 2011; Crittenden and Graybiel, 2011; Graybiel and Matsushima, 2023). It is therefore plausible that striosomal D_1_-MSNs may serve as an anatomical substrate responsible for the development of dystonia and OCD.

Notably, the network activity of the prefronto-striosomal pathway is responsive to modulation by incoming signals through mono-synaptic and/or multi-synaptic pathways that originate from diverse brain regions, including the thalamus, cerebellum, and brainstem. This indicates the potential for symptoms associated with dystonia and OCD to arise from functional impairments in any of the brain regions through the striatal striosome mechanism. Moreover, the output activities of striosomal D_1_-MSNs can be profoundly affected by their intricate interactions with a range of neurotransmitters, including gamma-aminobutyric acid (GABA), acetylcholine, and glutamate (Graybiel and Matsushima, 2023; Goto, 2025). Within the intricate striatal microcircuits, these neurotransmitter interactions foster a dynamic interplay that shapes the overall functionality of striosomal D_1_-MSNs (Graybiel and Matsushima, 2023; Goto, 2025). Consequently, considering dystonia and OCD as network disorders underscores the importance of gaining a comprehensive understanding of these dynamics, which may provide valuable insights into the pathophysiology of both conditions.

### 4.3 Therapeutic role of striosomal D_1_R signaling in dystonia OCD in humans

In the human striatum, D_1_Rs are heavily enriched in the striosomes, in contrast to their limited presence in the matrix compartment (Goto, 2023; Goto, 2025). This novel finding indicates that in humans, D_1_R-mediated signals are primarily processed through striosome-based circuits, and the processes of motor and mental action selection are primarily reliant on the activity of striosomal D_1_-MSNs (Goto, 2025). This specific compartmental distinction in D_1_R density enables the investigation of dopaminergic modulation targeting striosomal D_1_-MSNs (Matsumoto et al., 2022, 2024; Matsumoto and Goto, 2024). When exposed to low-dose L-DOPA, its D_1_-agonistic effects primarily act upon striosomal D_1_-MSNs, activating D_1_Rs to promote cAMP production, which in turn enhances striosomal D_1_-MSN activity (Figure 2B). The strategic concomitant use of low-dose CPZ may amplify the D_1_-agonistic effects of L-DOPA on striosomal D_1_-MSNs in a synergistic manner, (Matsumoto et al., 2022, 2024) as shown in Table 1. This is because low-dose D_2_ antagonists (e.g., CPZ) predominantly affect presynaptic D_2_Rs, known as D_2_ autoreceptors (D_2_ARs), located on the mesostriatal axon terminals, thereby increasing striatal dopamine release and activity (Figure 2B; Chen et al., 2020; Chakroun et al., 2023; Goto, 2025). The efficacy of this D_2_AR-mediated feedback mechanism depends on a balanced occupancy of both presynaptic and postsynaptic D_2_Rs by D_2_ antagonists within the striatum (Chen et al., 2020; Chakroun et al., 2023). Consequently, it is plausible that a combination of low-dose L-DOPA and CPZ could enhance striosomal D_1_R signaling, thereby restoring the proper functionality of the CTSC loops at the striatal level, which could lead to improvements in idiopathic dystonia and OCD. This implies that reduced striatal dopamine signaling may contribute to both dystonia and OC symptoms. This notion bears relevance to clinical observations indicating that dystonia can coexist with OCD in hypodopaminergic conditions, such as Parkinson’s disease (Mallet et al., 2002) and dopa-responsive dystonia (Antelmi et al., 2015). Also, a significant proportion of schizophrenia patients receiving high-dose antipsychotics (D_2_ antagonists) subsequently develop dystonia (Goto, 2025) and/or OCD (Biria et al., 2019) as a side effect. Notably, a recent study in rodents reveals that the effectiveness of high-dose antipsychotics is primarily linked to the modulation of D_1_-MSNs, rather than D_2_-MSNs, in the striatum (Yun et al., 2023).

### 4.4 Dystonia and OCD as syndromes associated with multiple etiologies

Dystonia and OCD are multifaceted conditions that occur due to a variety of etiological factors (Balint et al., 2018; Singh et al., 2023). An understanding of their complexities is essential for developing effective therapeutic interventions. It is important to note that repetitive stereotyped motor and cognitive symptoms can arise from either a paucity or an excess of striosomal D_1_-MSN activity (Amemori et al., 2011; Goto, 2025). Specific subgroups of patients may exhibit dystonia (Goto, 2025) and/or OCD (Crittenden et al., 2021) associated with heightened striosomal D_1_R signaling. In such instances, the antagonism of D_1_Rs, which can be achieved through the use of D_1_ antagonists (Goto, 2025) or high-dose D_2_ antagonists (Yun et al., 2023; Goto, 2025), may prove beneficial.

In the context of OCD, there is a concept that excessive dopamine signaling contributes to the condition, primarily due to the “phasic” release of dopamine within the CSTC (Wong et al., 2008; Sesia et al., 2013; Stein et al., 2019; Yin et al., 2023). According to this excessive dopamine hypothesis of OCD, dopamine receptor antagonists have been employed in treating OCD; however, their clinical efficacy remains limited, even at high doses, and their use carries a potential risk of serious side effects (Pittenger, 2021; van Roessel et al., 2023). In hypodopaminergic conditions, such as those induced by high-dose antipsychotic medications, a significant decrease in dopamine signaling can lead to an increased sensitivity of dopamine receptors to episodic dopamine surges. This phenomenon, known as dopamine (receptor) supersensitivity, is particularly attributed to the heightened activity of D_2_Rs coupling with adenosine A_2A_ receptors (Kostrzewa et al., 2018). These insights may elucidate the beneficial yet limited efficacy of dopamine antagonists in the treatment of OCD. For individuals presenting hypodopaminergic states, it is crucial to prioritize the enhancement of dopamine signaling as a core strategy for effectively addressing OCD. In these instances, the use of high-dose D_2_ or D_1_ antagonists may disrupt the logical order of treatment strategy and could lead to serious adverse effects over time, such as tardive syndrome (Goto, 2025). Based on the current findings, these considerations may also be relevant to the treatment of idiopathic dystonia.

Overall, it is imperative to recognize that both dystonia and OCD can manifest in conditions with either deficient or excessive dopamine signaling in the striatum. This understanding is crucial for determining the most suitable dopaminergic treatment for individual patients with dystonia or OCD. Furthermore, it may elucidate the reasons behind the often conflicting or unsatisfactory outcomes associated with conventional dopaminergic medications in the treatment of these challenging disorders.

## 5 Conclusion

This study presents compelling evidence that low-dose L-DOPA combined with CPZ can produce significant improvements in idiopathic dystonia and OCD by enhancing striatal (striosomal) D_1_R signaling. Our findings suggest a shared pathophysiology between these conditions, linked to striatal dysfunction due to dysregulated D_1_R activity in the striosomes. This insight contributes to the understanding of their pathophysiology and suggests that dopaminergic treatments aimed at restoring proper striosomal dopamine activity could serve as a practical approach for treating dystonia and OCD. However, the present study is limited by its relatively small participant pool, underscoring the need for further validation of the findings through research that utilizes a larger sample size and employs double-blinded, multi-center analyses, as well as comprehensive documentation of all adverse events that occur during the research process. Finally, we advocate for the advancement of clinical research focused on pharmacologically modulating striosomal D_1_R signaling to elucidate the intricate striatal mechanisms that govern the selection and execution of specific motor and mental actions, in both normative and pathological contexts. In pursuit of this objective, we express the strong expectation that advancements in high-resolution, cutting-edge *in vivo* brain imaging techniques, such as functional magnetic resonance imaging (fMRI) and positron emission tomography (PET), will provide researchers with dynamic insights into the functioning of the striatal striosome-matrix dopamine system in humans. These advanced imaging capabilities are anticipated to enhance our understanding of the intricate interplay between motor functions and cognitive processes, potentially leading to the development of innovative treatment strategies for debilitating basal ganglia disorders, including dystonia and OCD.

## Data Availability

The original contributions presented in this study are included in this article/Supplementary material, further inquiries can be directed to the corresponding author.

## Footnotes

1
https://omim.org/phenotypicSeries/PS128100

## Supplementary material

Supplementary Video 1 The case with Meige syndrome.

Supplementary Video 2 The case with cervical dystonia (horizontal type).

Supplementary Video 3 The case with cervical dystonia (antecollis).

Supplementary Video 4 The case with truncal dystonia.

Supplementary Video 5 The case with focal hand dystonia.
