# Supplementary material for: Therapeutic effects of striatal dopaminergic modulation on idiopathic dystonia and OCD in humans: insights from the striosome hypothesis
Source: Front Hum Neurosci. 2025 Aug 20;19:1621054. doi: 10.3389/fnhum.2025.1621054 (PMC12405262; doi:10.3389/fnhum.2025.1621054)
Supplement: Supplementary file 6 [file Table_1.docx]

**eTable 1.**　Dystonia subtypes and concurrent medications except for L-DOPA and chlorpromazine (CPZ) in L-DOPA, CPZ, and L-DOPA+CPZ groups.

| Patient No. | dystonia subtypes | Concurrent medications |
| --- | --- | --- |
| 1 | cervical & truncal dystonia | clonazepam |
| 2 | blepharospasm & oromandibular dystonia | clonazepam, etizolam, trazodone & diazepam |
| 3 | blepharospasm & oromandibular dystonia | clonazepam |
| 4 | cervical dystonia | clonazepam, zolpidem & eszopiclone |
| 5 | cervical dystonia | clonazepam |
| 6 | blepharospasm & oromandibular dystonia | trihexyphenidyl, eszopiclone, cloxazolam &lorazepam |

L-DOPA group

| Patient No. | dystonia subtypes | Concurrent medications |
| --- | --- | --- |
| 1 | blepharospasm | biperiden |
| 2 | cervical & hand dystonia | trihexyphenidyl & clonazepam |
| 3 | cervical dystonia | clonazepam |
| 4 | cervical & truncal dystonia | clonazepam & gabapentin |
| 5 | blepharospasm | lamotrigine & zolpidem |
| 6 | blepharospasm & oromandibular dystonia | clonazepam |

CPZ group

| Patient No. | dystonia subtypes | Concurrent medications |
| --- | --- | --- |
| 1 | blepharospasm & oromandibular dystonia | lorazepam, etizolam, ethylloflazepate & paroxetine |
| 2 | cervical dystonia | trihexyphenidyl, clonazepam & baclofen |
| 3 | blepharospasm　& cervical dystonia | zolpidem |
| 4 | cervical dystonia | pregabalin |
| 5 | cervical dystonia | pregabalin |
| 6 | blepharospasm & oromandibular dystonia | clonazepam |

L-DOPA+CPZ group

Abbreviations: L-DOPA; levodopa carbidopa hydrate, CPZ; chlorpromazine phenolphthalinate
