# Supplementary material for: Therapeutic effects of striatal dopaminergic modulation on idiopathic dystonia and OCD in humans: insights from the striosome hypothesis
Source: Front Hum Neurosci. 2025 Aug 20;19:1621054. doi: 10.3389/fnhum.2025.1621054 (PMC12405262; doi:10.3389/fnhum.2025.1621054)
Supplement: Supplementary file 7 [file Table_2.docx]

**eTable 2.** Dystonia subtypes and concurrent medications, except for L-DOPA and chlorpromazine, in 26 participants enrolled in a one-year follow-up study.

| Patient | dystonia subtypes | Concurrent medications |
| --- | --- | --- |
| No. |  |  |
| 1 | blepharospasm & oromandibular dystonia | lorazepam, etizolam, ethylloflazepate & paroxetine |
| 2 | cervical dystonia | trihexyphenidyl, clonazepam & baclofen |
| 3 | blepharospasm | none |
| 4 | blepharospasm & oromandibular dystonia | clonazepam |
| 5 | oromandibular dystonia | lamotrigine & duloxetine |
| 6 | oromandibular dystonia | none |
| 7 | oromandibular dystonia | etizolam, lorazepam & atomoxetine |
| 8 | cervical dystonia | pregabalin |
| 9 | cervical dystonia | pregabalin |
| 10 | cervical & oromandibular dystonia | clonazepam & zolpidem |
| 11 | cervical dystonia | none |
| 12 | cervical & truncal dystonia | lamotrigine & zolpidem |
| 13 | cervical & truncal dystonia | trihexyphenidyl & clonazepam |
| 14 | cervical & truncal dystonia | none |
| 15 | cervical & truncal dystonia | clonazepam & gabapentin |
| 16 | hand dystonia | pregabalin |
| 17 | blepharospasm & truncal dystonia | sertraline |
| 18 | lower limb dystonia | none |
| 19 | blepharospasm & oromandibular dystonia | clonazepam |
| 20 | blepharospasm & oromandibular dystonia | clonazepam, etizolam, trazodone & diazepam |
| 21 | cervical dystonia | clonazepam & biperiden |
| 22 | oromandibular dystonia | none |
| 23 | blepharospasm | none |
| 24 | cervical dystonia | sertraline & triazolam |
| 25 | hand dystonia | lamotrigine, clonazepam & duloxetine |
| 26 | lower limb dystonia | trihexyphenidyl, clonazepam & baclofen |
