# Supplementary material for: Therapeutic effects of striatal dopaminergic modulation on idiopathic dystonia and OCD in humans: insights from the striosome hypothesis
Source: Front Hum Neurosci. 2025 Aug 20;19:1621054. doi: 10.3389/fnhum.2025.1621054 (PMC12405262; doi:10.3389/fnhum.2025.1621054)
Supplement: Supplementary file 8 [file Table_3.docx]

**eTable 3.**　Obsessive–compulsive disorder subtypes based on Yale Brown Obsessive-Compulsive Scale Symptom Checklist in L-DOPA, CPZ, and L-DOPA+CPZ groups.

| Subgroups | | CPZ group | | | | | |  | LDOPA group | | | | | |  | LDOPA＋CPZ group | | | | | |
| --- | --- | --- | --- | --- | --- | --- | --- | --- | --- | --- | --- | --- | --- | --- | --- | --- | --- | --- | --- | --- | --- |
| Patient No. | | 1 | 2 | 3 | 4 | 5 | 6 |  | 1 | 2 | 3 | 4 | 5 | 6 |  | 1 | 2 | 3 | 4 | 5 | 6 |
| Age (years) | | 20s | 50s | 60s | 50s | 50s | 40s |  | 60s | 60s | 50s | 80s | 50s | 70s |  | 70s | 30s | 70s | 70s | 80s | 70s |
| Disease duration (years) | | 1 | 8 | 7 | 16 | 5 | 5 |  | 7 | 3 | 24 | 13 | 9 | 13 |  | 6 | 13 | 17 | 4 | 3 | 12 |
| **Obsessive-compulsive disorder subtype**s | AGGRESSIVE OBSESSIONS | S | S | S | S | S | S |  | N | S | N | S | S | S |  | S | N | S | S | N | N |
|  | CONTAMINATION OBSESSIONS | N | S | S | S | S | N |  | S | S | S | S | N | S |  | S | N | S | S | S | N |
|  | SEXUAL OBSESSIONS | N | N | N | N | N | N |  | N | N | N | N | N | N |  | S | N | S | N | N | N |
|  | HOARDING/SAVING OBSESSIONS | N | S | N | S | S | N |  | N | S | N | N | S | S |  | S | N | S | S | N | N |
|  | RELIGIOUS OBSESSIONS | N | S | N | S | S | N |  | S | S | N | N | N | N |  | S | N | S | S | S | N |
|  | OBSESSION WITH NEED FOR SYMMETRY OR EXACTNESS | N | N | S | S | N | N |  | S | S | N | N | N | N |  | S | N | N | N | S | N |
|  | MISCELLANEOUS OBSESSIONS | S | S | S | S | S | S |  | S | S | S | S | S | S |  | S | S | S | S | S | S |
|  | SOMATIC OBSESSIONS | S | S | S | S | S | N |  | S | S | S | S | S | S |  | S | N | S | S | S | S |
|  | CLEANING/WASHING COMPULSIONS | N | N | S | S | S | N |  | S | N | S | N | N | N |  | S | N | N | S | S | N |
|  | CHECKING COMPULSIONS | S | S | N | S | S | S |  | S | S | N | S | S | N |  | S | S | S | S | S | N |
|  | REPEATING RITUALS | N | S | S | S | N | N |  | N | S | N | S | S | N |  | S | N | S | S | N | N |
|  | COUNTING COMPULSIONS | N | N | S | S | S | N |  | N | N | N | N | N | N |  | S | N | S | N | N | N |
|  | ORDERING/ARRANGING COMPULSIONS | N | N | N | S | N | N |  | S | N | N | S | N | N |  | N | N | N | S | S | N |
|  | HOARDING/COLLECTING COMPULSIONS | N | N | N | S | N | N |  | N | S | N | N | N | S |  | S | N | S | N | N | N |
|  | MISCELLANEOUS COMPULSIONS | S | S | S | S | S | N |  | S | S | N | N | N | S |  | S | N | S | S | S | S |

Abbreviations: CPZ; chlorpromazine phenolphthalinate, L-DOPA; levodopa carbidopa hydrate, S; symptomstic, N; no symptom
