# Supplementary material for: Therapeutic effects of striatal dopaminergic modulation on idiopathic dystonia and OCD in humans: insights from the striosome hypothesis
Source: Front Hum Neurosci. 2025 Aug 20;19:1621054. doi: 10.3389/fnhum.2025.1621054 (PMC12405262; doi:10.3389/fnhum.2025.1621054)
Supplement: Supplementary file 9 [file Table_4.docx]

**eTable 4.**　Obsessive–compulsive disorder subtypes based on Yale Brown Obsessive-Compulsive Scale Symptom Checklist in 26 participants enrolled in a one-year follow-up study.

| Patient No. | | 1 | 2 | 3 | 4 | 5 | 6 | 7 | 8 | 9 | 10 | 11 | 12 | 13 | 14 | 15 | 16 | 17 | 18 | 19 | 20 | 21 | 22 | 23 | 24 | 25 | 26 |
| --- | --- | --- | --- | --- | --- | --- | --- | --- | --- | --- | --- | --- | --- | --- | --- | --- | --- | --- | --- | --- | --- | --- | --- | --- | --- | --- | --- |
| Age (years) | | 70s | 70s | 30s | 70s | 30s | 60s | 40s | 70s | 80s | 40s | 80s | 50s | 40s | 50s | 50s | 40s | 30s | 40s | 50s | 70s | 30s | 30s | 60s | 50s | 40s | 50s |
| Disease duration (years) | | 6 | 17 | 1 | 12 | 9 | 5 | 2 | 4 | 3 | 4 | 0 | 6 | 6 | 1 | 17 | 20 | 1 | 5 | 25 | 4 | 13 | 9 | 13 | 5 | 19 | 15 |
| Obsessive-compulsive disorder subtypes | AGGRESSIVE OBSESSIONS | S | S | S | N | N | N | S | S | N | S | N | S | S | S | S | S | S | S | N | S | S | N | N | S | S | S |
|  | CONTAMINATION OBSESSIONS | S | S | N | N | S | N | S | S | S | N | N | S | N | S | S | N | N | N | S | S | N | S | N | S | N | N |
|  | SEXUAL OBSESSIONS | S | S | N | N | N | N | S | N | N | N | N | N | N | N | N | N | N | N | N | N | N | N | N | N | N | N |
|  | HOARDING/SAVING OBSESSIONS | S | S | S | N | N | N | S | S | N | N | N | S | N | N | S | S | N | N | N | S | N | N | N | S | S | S |
|  | RELIGIOUS OBSESSIONS | S | S | N | N | N | N | S | S | S | N | N | S | N | N | S | N | N | N | N | S | N | S | N | N | N | S |
|  | OBSESSION WITH NEED FOR SYMMETRY OR EXACTNESS | S | N | N | N | N | N | N | N | S | N | S | N | N | N | S | N | N | N | N | S | N | N | N | N | S | N |
|  | MISCELLANEOUS OBSESSIONS | S | S | S | S | N | S | S | S | S | S | S | S | S | S | S | S | S | N | S | S | N | S | N | S | S | S |
|  | SOMATIC OBSESSIONS | S | S | S | S | S | S | S | S | S | N | S | S | S | S | S | S | S | S | S | S | N | S | S | S | S | S |
|  | CLEANING/WASHING COMPULSIONS | S | N | S | N | S | N | N | S | S | N | S | S | N | N | S | N | N | S | S | N | N | S | N | N | S | N |
|  | CHECKING COMPULSIONS | S | S | S | N | N | S | S | S | S | S | S | S | S | S | S | S | N | S | N | S | S | S | S | S | S | S |
|  | REPEATING RITUALS | S | S | S | N | S | S | N | S | N | N | S | N | N | N | S | N | N | N | N | S | N | N | N | N | N | N |
|  | COUNTING COMPULSIONS | S | S | N | N | S | N | N | N | N | N | N | S | N | N | S | N | N | N | N | N | N | N | N | N | N | N |
|  | ORDERING/ARRANGING COMPULSIONS | N | N | N | N | N | N | N | S | S | N | S | N | N | N | S | N | N | N | N | N | N | N | S | N | S | S |
|  | HOARDING/COLLECTING COMPULSIONS | S | S | S | N | N | N | N | N | N | N | S | N | N | N | S | N | N | N | N | S | N | N | N | N | N | N |
|  | MISCELLANEOUS COMPULSIONS | S | S | S | S | N | N | S | S | S | N | S | S | N | N | S | S | N | N | N | S | N | S | S | N | N | S |

Abbreviations: CPZ; chlorpromazine phenolphthalinate, L-DOPA; levodopa carbidopa hydrate, S; symptomstic, N; no symptom
